# Supplementary material for: How flagellar glycosylation of the phytopathogenic bacterium Pseudomonas amygdali pv. tabaci 6605 affects transport and deposition in saturated sandy porous media
Source: Appl Environ Microbiol. 2026 Jan 12;92(2):e02111-25. doi: 10.1128/aem.02111-25 (PMC12915359; doi:10.1128/aem.02111-25)

# “How flagella glycosylation of the phytopathogenic bacteria *Pseudomonas amygdali* pv. tabaci 6605 affect transport and deposition in saturated sandy porous media”

Submitted to Journal

**“**[**Applied**](https://link.springer.com/journal/11356?IFA) **and Environmental Microbiology”**

**Xin Zheng^1,2^, Mounia Achak^3^, Edvina Lamy^1,5*^ and Yannick Rossez^2,4,5*^**

^1^ Sorbonne université, Université de Technologie de Compiègne, UTC/ESCOM, EA 4297 TIMR, Centre de recherche Royallieu, CS 60 319, 60203 Compiègne cedex, France

^2^ Université de technologie de Compiègne, UPJV, UMR CNRS 7025, Enzyme and Cell Engineering, Centre de recherche Royallieu, CS 60319, 60203 Compiègne Cedex, France

^3^ Mohammed VI Polytechnic University (UM6P), Chemical and Biochemical Sciences, Green Process Engineering, Benguerir 43150, Morocco

^4^ Present address: Univ. Lille, CNRS, UMR 8576 - UGSF - Unité de Glycobiologie Structurale et Fonctionnelle, Lille, France

^5^ These authors jointly supervised this work: Yannick Rossez, Edvina Lamy

*Correspondence: [edvina.lamy@utc.fr](mailto:edvina.lamy@utc.fr), yannick.rossez@univ-lille.fr

**Supplementary Material**

**S1** Process of column transport experiments

Two filter papers were used at the inlet and outlet of the column to make sure the solution came out without fine sand particles during the transport experiment. Before every experiment, the pump, tubes, and other column components were sterilized with bleach and 15 min of UV. Other materials were sterilized using an autoclave. The empty column with pipes, two caps, and two filter papers was weighted, and the mass was noted as M_0_ (g). The sand was inserted in successive layers into the column to assure homogenous distribution, and both column and sand were weighted and noted as M_1_ (g). The mass of sand was estimated as:

| $M_{s}=M_{1}-M_{0}$ | (1) |
| --- | --- |

and the sand bulk density (*ρ_b_*) was calculated by dividing the dry mass of the sand (*Ms*) by the volume of the column (*Vc*):

| $\rho_{b}={M_{S}}/{V_{c}}$ | (2) |
| --- | --- |

The porosity ($\varepsilon)$ was then estimated by the sand bulk density as follows:

| $\varepsilon=\left( 1-\rho_{b}/2.65 \right)\times100\%$ | (3) |
| --- | --- |

The column was flushed upward using a background solution of NaCl (0.1 mmol/L), then the column was rinsed using the background solution reversed, and at the same time, the conductivity of the effluent solution was continuously measured at the column outlet until this parameter reached a stable value. Then, the column fully saturated with the background solution was weighed, and this mass was noted as M_2_ (g). Assuming that water density is 1 g/cm^3^, the volume occupied by water (*V_w_*) was estimated as:

| $V_{w}=M_{2}-M_{1}$ | (4) |
| --- | --- |

The saturation degree (*S*) could then be described as follows:

| $S=V_{w}/V_{p}$ | (5) |
| --- | --- |

here *V_p_* is the volume of the pores, calculated as

| ${V_{p}= V}_{c}\times\varepsilon$ | (6) |
| --- | --- |

For bacteria injection experiments, 20 mL of bacterial suspension was injected into each column (Figure 1). The breakthrough curve of each strain was obtained by plotting bacterial concentration as a function of pore volume *V*/*V*_0_. Bacteria concentration was deduced by measuring the absorbance of effluent at OD_600_ at the column outlet. The bacterial suspension was flushed with a background solution (0.1 mmol/L NaCl solution) until the absorbance of the effluent returned to the baseline level.


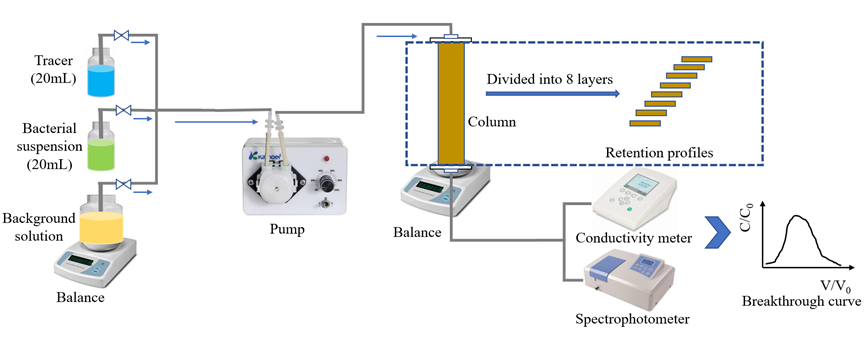


**Fig. 1** Schematic of column experiments.

**S2** Experimental conditions of *P. amygdali* pv. tabaci transport experiments under saturated conditions

| **Bacterial strains** | **Replicate** | **Saturated degree (%)** | **C_0_**  **(CFU/ml)** | **Porosity**  **(%)** | **Bulk density (g/cm^3^)** | **Pulse time**  **(min)** | **Darcy velocity (cm/min)** |
| --- | --- | --- | --- | --- | --- | --- | --- |
|  |  |  |  |  |  |  |  |
| WT | 1 | 98.0 | 5.30E+08 | 37.45 | 1.66 | 2.47 | 0.893 |
|  | 2 | 97.0 | 5.00E+08 | 37.22 | 1.66 | 2.45 | 0.9 |
|  | 3 | 98.2 | 4.67E+08 | 38.57 | 1.63 | 2.17 | 1.017 |
| Δ*fliC* | 1 | 99.7 | 5.67E+08 | 38.43 | 1.63 | 2.23 | 0.987 |
|  | 2 | 96.3 | 5.33E+08 | 38.67 | 1.63 | 2.20 | 1.002 |
|  | 3 | 96.9 | 5.73E+08 | 38.72 | 1.62 | 2.18 | 1.01 |
| Δ***fgt1*** | 1 | 97.1 | 5.43E+08 | 38.96 | 1.62 | 2.20 | 1.002 |
|  | 2 | 98.2 | 5.60E+08 | 38.82 | 1.62 | 2.23 | 0.987 |
|  | 3 | 97.2 | 5.53E+08 | 38.94 | 1.65 | 2.183 | 1.01 |
| Δ***fgt2*** | 1 | 98.6 | 5.83E+08 | 38.81 | 1.62 | 2.23 | 0.987 |
|  | 2 | 99.5 | 5.63E+08 | 38.93 | 1.62 | 2.20 | 1.002 |
|  | 3 | 99.5 | 6.03E+08 | 38.8 | 1.62 | 2.25 | 0.98 |

**S3** Fitted parameters (replicates) for *Pseudomonas amygdali* pv. tabaci through saturated columns using MIM model in HYDRUS-1D code

| **Bacterial strains** | **Replicate** | ***λ* (cm)** | ***θ_m_/ θ*** | ***K_att_* (min^-1^)** | ***K_d_* (min^-1^)** | ***K_str_* (min^-1^)** | ***R^2^*（BTC）** |
| --- | --- | --- | --- | --- | --- | --- | --- |
| WT | 1 | 0.68 | 0.62 | 4.13E-02 | 4.42E-01 | 1.77 | 0.98603 |
|  |  | (0.08) ^a^ | (0.009) | 1.21E-02 | 4.19E-01 | 2.66E-01 |  |
|  | 2 | 0.24 | 0.64 | 4.02E-01 | 1.65E-02 | 3.88E-02 | 0.95463 |
|  |  | (0.13) | (0.007) | 1.53E-01 | 1.50E-02 | 8.17E-01 |  |
|  | 3 | 0.39 | 0.65 | 8.59E-02 | 7.08E-01 | 1.40E+00 | 0.99716 |
|  |  | (0.05) | (0.006) | 3.38E-02 | 2.28E-01 | 2.53E-02 |  |
| Mean values |  | 0.44 | 0.64 | 0.18 | 0.39 | 1.07 | 0.979 |
|  |  | (0.2237) ^b^ | 0.0172 | 0.1965 | 0.3488 | 0.9103 | 0.0221 |
| Δ*fliC* | 1 | 0.25 | 0.65 | 1.09E-01 | 1.87E+00 | 8.99E-01 | 0.99197 |
|  |  | (0.09) | (0.02) | 2.89E-01 | 2.53E+00 | 5.56E-02 |  |
|  | 2 | 0.34 | 0.66 | 1.95E-01 | 4.42E+00 | 8.02E-01 | 0.96517 |
|  |  | (0.3) | (0.052) | 2.71E+00 | 3.86E+01 | 1.85E-01 |  |
|  | 3 | 0.32 | 0.66 | 2.45E-01 | 2.90E+00 | 8.79E-01 | 0.99669 |
|  |  | (0.06) | (0.016) | 4.92E-01 | 2.90E+00 | 4.97E-02 |  |
| Mean values |  | 0.30 | 0.66 | 0.18 | 3.06 | 0.86 | 0.985 |
|  |  | 0.0470 | 0.0049 | 0.0690 | 1.2817 | 0.0513 | 0.0170 |
| Δ*fgt1* | 1 | 0.28 | 0.66 | 1.83E-01 | 2.33E+00 | 2.34E+00 | 0.99251 |
|  |  | (0.08) | (0.03) | 4.26E-01 | 2.73E+00 | 7.95E-02 |  |
|  | 2 | 0.17 | 0.64 | 2.64E-01 | 2.38E+00 | 2.52E+00 | 0.99817 |
|  |  | (0.04) | (0.007) | 1.66E-01 | 7.54E-01 | 3.73E-02 |  |
|  | 3 | 0.25 | 0.66 | 9.89E-02 | 1.44E+00 | 1.27E+00 | 0.99788 |
|  |  | (0.05) | (0.008) | 1.05E-01 | 8.24E-01 | 2.06E-02 |  |
| Mean values |  | 0.23 | 0.66 | 0.18 | 2.05 | 2.04 | 0.996 |
|  |  | 0.0569 | 0.0132 | 0.0827 | 0.5274 | 0.6729 | 0.0032 |
| Δ*fgt2* | 1 | 0.27 | 0.68 | 7.62E-02 | 1.30E+00 | 1.18E+00 | 0.9977 |
|  |  | (0.05) | (0.03) | 9.24E-02 | 8.94E-01 | 2.13E-02 |  |
|  | 2 | 0.29 | 0.68 | 6.71E-02 | 1.27E+00 | 1.15E+00 | 0.99751 |
|  |  | (0.05) | (0.009) | 9.43E-02 | 1.03E+00 | 2.22E-02 |  |
|  | 3 | 0.24 | 0.66 | 9.09E-02 | 1.29E+00 | 1.27E+00 | 0.99776 |
|  |  | (0.04) | (0.007) | 8.42E-02 | 6.92E-01 | 2.20E-02 |  |
| Mean values |  | 0.27 | 0.68 | 0.08 | 1.29 | 1.20 | 0.998 |
|  |  | 0.0256 | 0.0102 | 0.0120 | 0.0137 | 0.0626 | 0.0001 |

^a^ the values given were the standard error coefficients (S.E.Coeff) obtained from HYDRUS-1D code, ^b^ the values given parentheses were the standard deviation

**S4** Gel electrophoresis of purified flagella from *Pseudomonas amygdali* pv. tabaci WT and glycosylation-defective strains


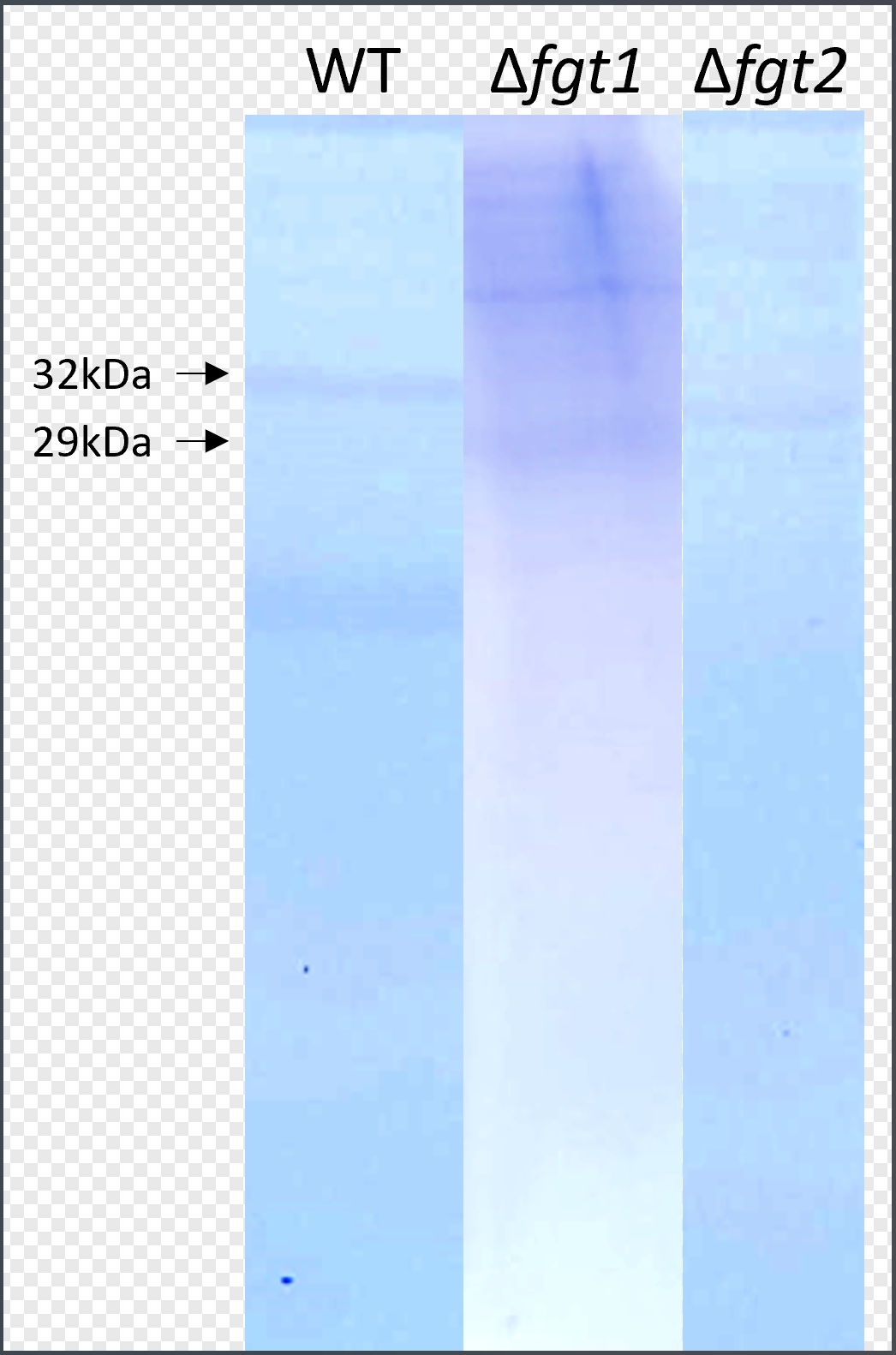

Supplement: Supplemental material — Experimental process, experimental conditions, and specific fitted parameters. [file aem.02111-25-s0001.docx]
